# Using long term mortality to determine which perioperative risk factors of mortality following hip and knee replacement may be causal

**DOI:** 10.1038/s41598-018-33314-0

**Published:** 2018-10-09

**Authors:** Linda P. Hunt, Michael R. Whitehouse, Peter W. Howard, Yoav Ben-Shlomo, Ashley W. Blom

**Affiliations:** 1 Musculoskeletal Research Unit, Translational Health Sciences, Bristol Medical School, 1st Floor Learning & Research Building, Southmead Hospital, Bristol, BS10 5NB United Kingdom; 2 National Institute for Health Research Bristol Biomedical Research Centre, University of Bristol, Bristol, United Kingdom; 3 Department of Trauma and Orthopaedics, Royal Derby Hospital, Uttoxeter Road, Derby, DE22 3NE United Kingdom; 4 Population Health Sciences, Bristol Medical School, Canynge Hall, 39 Whatley Road, Bristol, BS8 2PS United Kingdom

## Abstract

Observational studies have identified surgical factors that are associated with a reduced risk of mortality after joint replacement. It is not clear whether these are causal or reflect patient selection. Data on the first primary hip (n = 424,156) and knee replacements (n = 469,989) performed for osteoarthritis in the National Joint Registry were analysed. Flexible parametric survival modelling was used to determine if risk factors for mortality in the perioperative period persisted. To explore selection bias, standardised mortality ratios were calculated for all-cause, respiratory and smoking related cancer mortality using population rates. Selection was apparent for hip resurfacing, combined spinal and general anaesthetic and unicondylar knee implants; reduced mortality was observed for many years for both all and other causes of mortality with a waning effect. Mechanical thromboprophylaxis was also suggestive of selection although patients receiving aspirin had sustained reduced mortality, possibly due to to a cardioprotective effect. Posterior approach for hips was ambiguous with a possible causal component. Spinal anaesthesia was suggestive of a causal effect. We are reliant on observational data when it is not feasible to undertake randomised trials. Our approach of looking at long term mortality risks for perioperative interventions provides further insights to differentiate causal interventions from selection. We recommend the use of aspirin chemothromboprophylaxis, the posterior approach and spinal anaesthetic in total hip replacement due to the apparent causal effect on reduced mortality.

## Introduction

Total hip replacement (THR), total knee replacement (TKR) and unicompartmental knee replacement (UKR) cause short-term increases in mortality persisting for 90-days in hips^1^ and 45-days in knees^2^. Observational studies have identified surgical-related factors associated with decreased mortality. For hip replacement, these included posterior approach, thromboprophylaxis, spinal anaesthetic^1^ and resurfacing hip replacement^3^. For knee replacement, unicompartmental knee replacement was associated^2,4^.

Surgical-related exposures may be confounded by indication despite statistical adjustment. Randomised controlled trials are the “gold standard” for inferring causality but are unfeasible when the primary outcome is rare. Patients undergoing joint replacement are pre-selected as fit enough to undergo surgery and hence are healthier than the general population. The overall mortality in this population is less than expected using general population mortality rates, but over time this reduced risk diminishes^5^. Knowledge of the cause of death is helpful as health selection would be associated with reduced mortality from respiratory and smoking-related cancers, causes unlikely to be influenced by any perioperative surgery-related factor.

Our aim was to determine whether “protective” factors previously identified may be due to selection by examining extended follow-up cause-specific linked mortality data. We hypothesised that (i) truly causal interventions which only influence the perioperative period would show an acute short-term reduction in mortality. However, conditional on survival to the end of this period, the subsequent mortality patterns should be the same for both exposed and unexposed groups. We defined the perioperative period as being 90 days as this is the timepoint at which we have previously shown the increased risk of perioperative mortality associated with the surgical intervention returns to baseline^1^. (ii) If healthier participants were selected for the intervention, this group would have a persistently lower mortality at all time periods, that was unlikely to be explicable by the intervention, though over time mortality risks would converge towards that seen in the general population due to attenuation. (iii) There may be a causal benefit of the intervention but there is also selection of which patients receive this. In this case, it may not be possible to differentiate causality from selection.

## Methods

### Patients and Data Sources

539,372 and 589,028 linkable primary THRs and TKRs in the National Joint Registry of England and Wales (NJR), undertaken April 2003 to December 2012 inclusive and reported in the 10^th^ Annual Report^6^ were analysed. Dates of death were obtained from NHS Personal Demographics Service on 23rd February 2013. We excluded 261 THRs and 243 TKRs because the NHS number was untraceable, consent had been withdrawn or for ambiguous age or gender and a further 6,182 THRs (3,091 patients) and 15,142 TKRs (7,571 patients) with simultaneous bilateral operations. 479,191 THRs and 550,787 TKRs had osteoarthritis (OA) as the only indication for surgery; these two groups were analysed separately.

Patients often had other hip or knee procedures recorded, making it difficult to describe mortality associated with one incident procedure. Furthermore, when left and right joints were replaced at different times, any subsequent death would be included twice. After exploring several strategies, analysis was based on the first primary hip or knee procedure reported in the NJR; 424,156 first THRs and 469,989 first TKRs, of which 33,759 and 36,003 respectively died on/before our censoring date (31^st^ December 2012), as described previously^7^. For those who died, we obtained the main causes of death (ICD 10) from the Office for National Statistics (ONS) via NJR linkage to the patient’s Hospital Episode Statistics (HES) identifier, thereby excluding patients in the NJR with no inpatient HES records up to the end of November 2012. The latter included patients with no NHS-funded procedures recorded in the NJR, or with procedures performed only in Wales. 332,734 of the patients undergoing hip replacement (26,766 deaths) and 384,291 of the patients undergoing knee replacement (29,802 deaths) could be linked^7^.

### Exposures

Surgical related interventions found to be important in our earlier short-term mortality publications: posterior surgical approach, thromboprophylaxis, spinal anaesthetic, resurfacing hip replacement (for hip replacement)^1^ and unicompartmental knee replacement^2^.

### Other covariates

We also modelled age, gender, year of primary operation (2003–2005, 2006–2008, 2009–2012) and ASA grade. Where available, each procedure was linked to the patient’s HES inpatient records over a 5-year period prior to the operation date which were used to compute area deprivation quintiles^8–10^ and Charlson co-morbidity^1,2^. Comorbidity analysis was restricted to primary operations performed prior to the end of November 2012 as HES records beyond this date were not available. BMI was analysed separately as data were incomplete and not recorded in the early phase of NJR. Variable coding and frequency counts are shown in Supplementary Material Tables S1 and S2.

### Statistical Methods

Two complementary approaches were adopted (i) an internal and (ii) an external comparison. The internal comparison used proportional hazards regression models to model the time to death *from any cause* in the presence of censoring (see Supplementary Material Text S1). To capture changing hazard ratios over time, we used flexible parametric survival modelling (FPM)^11,12^ implemented in Stata^13,14^. We first sought parsimonious models with just gender and (continuous) age at operation (as four restricted cubic splines)^12^. We assessed time-varying effects of gender and age by adding appropriate terms to the model to represent these effects, assessing the changes with likelihood ratio tests and examination of Akaike and Bayesian information criteria (AIC and BIC respectively), giving preference to the former. We then added other risk-factor variables to the model, using a series of 0/1 indicators for each (Supplementary Material Tables S1 and S2). The effects of each risk factor on the HRs were assessed, adjusting for age and gender, and whether their effects changed with time. A final multivariable model was constructed of all surgical risk factors, plus age, gender, ASA and year of surgery. Adjusted HRs for each risk factor were plotted. Further models additionally adjusted for comorbidity and quintiles of area deprivation.

The external comparison compared the observed mortality patterns for exposures in relation to expected mortality using national rates. In contrast to the internal comparison, above, this analysis looked at one risk factor at a time, conditioning on contemporaneous age group and sex. To test for differing patterns of mortality over time we calculated Standardised Mortality Ratios (SMRs) by time interval (0–90 days, 90 days–1 year, 3–5 years, 5–7 years and 7 years+) from the primary operation by dividing the observed numbers of deaths (O) in each of these intervals by the expected numbers (E). The latter were calculated from ONS age/sex mid-year populations^15^ and relevant deaths^16^ for England and Wales. We did this with respect to (i) all cause deaths (ii) respiratory system deaths (all ICD-10 ‘J’ codes) and (iii) deaths from cancers that were believed to be related to smoking (see Supplementary Material Text S2). We also explored cardiovascular deaths, but these results are not shown for simplicity. These cause-specific analyses could only be performed for the subsets of patients with associated HES records (332,724 hips; 384,291 knees). We plotted the temporal changes in (logged) SMR with time (see Supplementary Material Fig. S1), exploring the observed patterns with Poisson regression models, with the expected number of deaths as the offsets. *Excluding the 90-day period deaths post operation*, the remaining intervals were coded: 90-days to 1 year = 0, 1–3 years = 1, 3 to 5 years = 2, 5 to 7 years = 3, 7 years plus = 4. The effects of the risk factor was assessed by fitting a group effect allowing for a common slope. Temporal divergence/convergence was tested by including a group by time period interaction. We hypothesised that if there were or were not group differences due to different intercepts but we observed the same slope, this would be suggestive of a causal effect. If there was a difference in the slopes, regardless of whether there was a difference or not in the intercept, this would represent a selection effect and be most likely observed as an improved SMR in the early period which abated with time as the benefit of the selection effect was reduced.

### Consent

Patients consent to participate was obtained at the point of data capture by the NJR. As this was an analysis of anonymised routinely collected, no separate ethical approval was required.

## Results

In modelling the baseline hazard for the internal model, 4 and 3 ‘knots’ (i.e. degrees of freedom (df) = 5 and 4) sufficiently captured the hip and knee patterns seen in our earlier analyses^1,2^; i.e. short term increases that subsided by 90 and 45-days, respectively, thereafter increasing with time, reflecting normal mortality.

The effects of both age and gender on the hazard rate were time-varying; Supplementary Material Fig. S2 details how they were modelled, the magnitude of their effects and illustrates that the estimated cumulative mortality fitted the observed mortality well. Supplementary Material Fig. S3 details the results from adding the other surgical covariates. Supplementary Material Figs S4 and S5 document additional analyses including bearing surface and grouped BMI.

### Posterior approach

**Figure 1:**
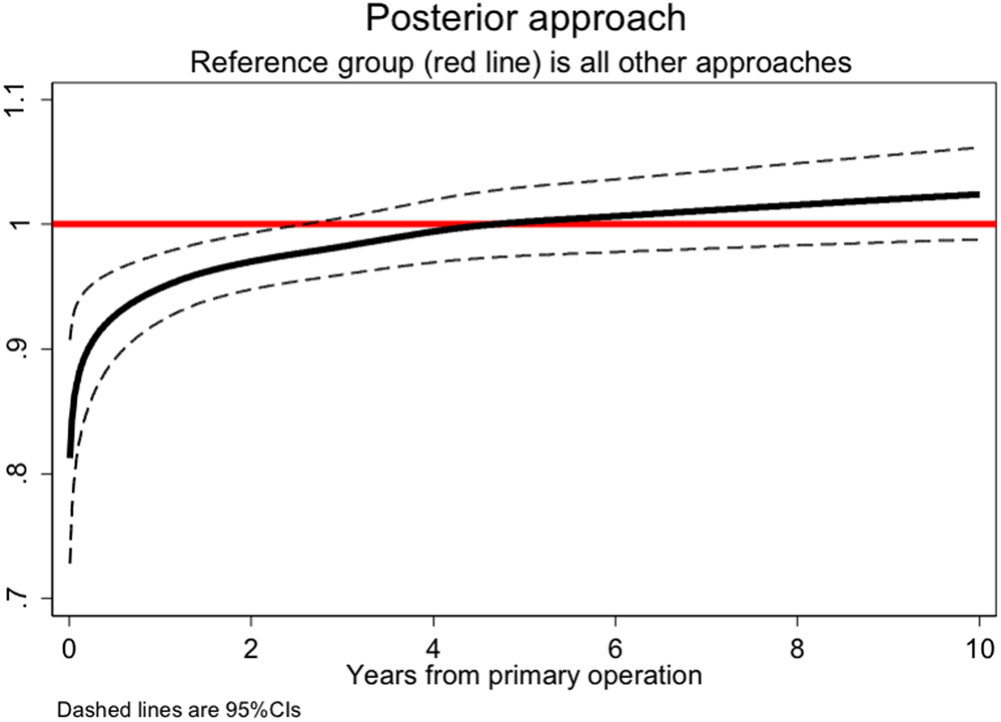
Hazard rates ratios (with pointwise 95% CIs) associated with a posterior approach for hips (adjusting for time varying effects of age, gender, year of operation, ASA, mechanical and chemical thromboprophylaxis, anaesthetic used and implant type).

The internal comparison for all-cause mortality showed a significant time-varying effect of the posterior approach compared with ‘other’ approaches (p < 0.001, likelihood ratio test); there was an attenuation of the protective hazard ratio by 2 years (Fig. 1). The external comparison for all-cause mortality found a group difference, the biggest difference was in the earliest period after which the SMRs were fairly similar for both groups, but slightly lower for the posterior approach (Table 1). There was no evidence of selection as smoking-related cancer mortality was almost identical across the exposure groups but far less than expected due to the population being selected for an elective operation. Respiratory mortality was less in the posterior approach group (p = 0.05).

**Table 1:** Comparison of SMRs between posterior approach and other surgical approaches for hips. (Ratios of observed (O) to expected (E) numbers of deaths obtained from national rates [with 95% CI shown in parentheses]).

HIP PRIMARIES	Main cause of death (ICD10)					
	(i) All causes of death		(ii) Respiratory system (all ‘J’ codes)		(iii) ‘Cancers related to smoking’*	
	Posterior (n = 218,566)	Other approaches (n = 205,590)	Posterior (n = 168,674)	Other approaches (n = 164,060)	Posterior (n = 168,674)	Other approaches (n = 164,060)
	O/E	O/E	O/E	O/E	O/E	O/E
All deaths	13,931/24,662.3 = 0.56 [0.56–0.57]	19,828/32,457.6 = 0.61 [0.60–0.62]	1,129/2,751.0 = 0.41 [0.39–0.44]	1,799/3,806.1 = 0.47 [0.45–0.50]	2,012/2,721.7 = 0.74 [0.71–0.77]	2,618/3,530.9 = 0.74 [0.71–0.77]
**Deaths by time interval post primary:**						
Within 90 d	798/1,380.0 = 0.58 [0.54–0.62]	1,053/1,504.7 = 0.70 [0.66–0.74]	50/154.6 = 0.32 [0.24–0.43]	72/173.8 = 0.41 [0.32–0.52]	37/170.9 = 0.22 [0.15–0.30]	34/183.8 = 0.18 [0.13–0.26]
90 d to 1 y	1,585/4,023.5 = 0.39 [0.37–0.41]	1,906/4,500.8 = 0.42 [0.40–0.44]	103/452.9 = 0.23 [0.19–0.28]	145/524.0 0.28 [0.23–0.33]	263/489.5 = 0.54 [0.47–0.61]	299/540.9 = 0.55 [0.49–0.62]
1 y to 3 y	4,557/9,025.6 = 0.50 [0.49–0.52]	5,652/10,877.6 = 0.52 [0.51–0.53]	343/1,016.8 = 0.34 [0.30–0.37]	464/1,279.0 = 0.36 [0.33–0.40]	776/1,029.6 = 0.75 [0.70–0.81]	867/1,236.2 = 0.70 [0.66–0.75]
3 y to 5 y	3,884/6,110.2 = 0.64 [0.62–0.66]	5,646/8,404.2 = 0.67 [0.65–0.69]	358/680.0 = 0.53 [0.47–0.58]	537/987.8 = 0.54 [0.50–0.59]	555/638.7 = 0.87 [0.80–0.94]	795/881.3 = 0.90 [0.84–0.97]
5 y to 7 y	2,258/3,110.2 = 0.73 [0.70–0.76]	3,786/5,078.3 = 0.75 [0.72–0.77]	195/336.7 = 0.58 [0.50–0.67]	385/593.5 = 0.65 [0.59–0.72]	296/299.7 = 0.99 [0.88–1.11]	443/494.7 = 0.90 [0.81–0.98]
7 y+	849/1,012.8 = 0.84 [0.78–0.90]	1,785/2,092.0 = 0.85 [0.81–0.89]	80/110.0 = 0.73 [0.58–0.91]	196/248.1 = 0.79 [0.68–0.91]	85/93.2 = 0.91 [0.73–1.13]	180/194.0 = 0.93 [0.80–1.07]
Post 90-day modelling: Group x period interaction; Group difference**	**Posterior vs. other:** P = 0.195; P = 0.001		**Posterior vs. other:** P = 0.548; P = 0.045		**Posterior vs. other:** P = 0.550; P = 0.340	

*See text for details;

**Assessed in the absence of a significant group x period interaction (but assuming a common slope).

### Mechanical thromboprophylaxis

**Figure 2:**
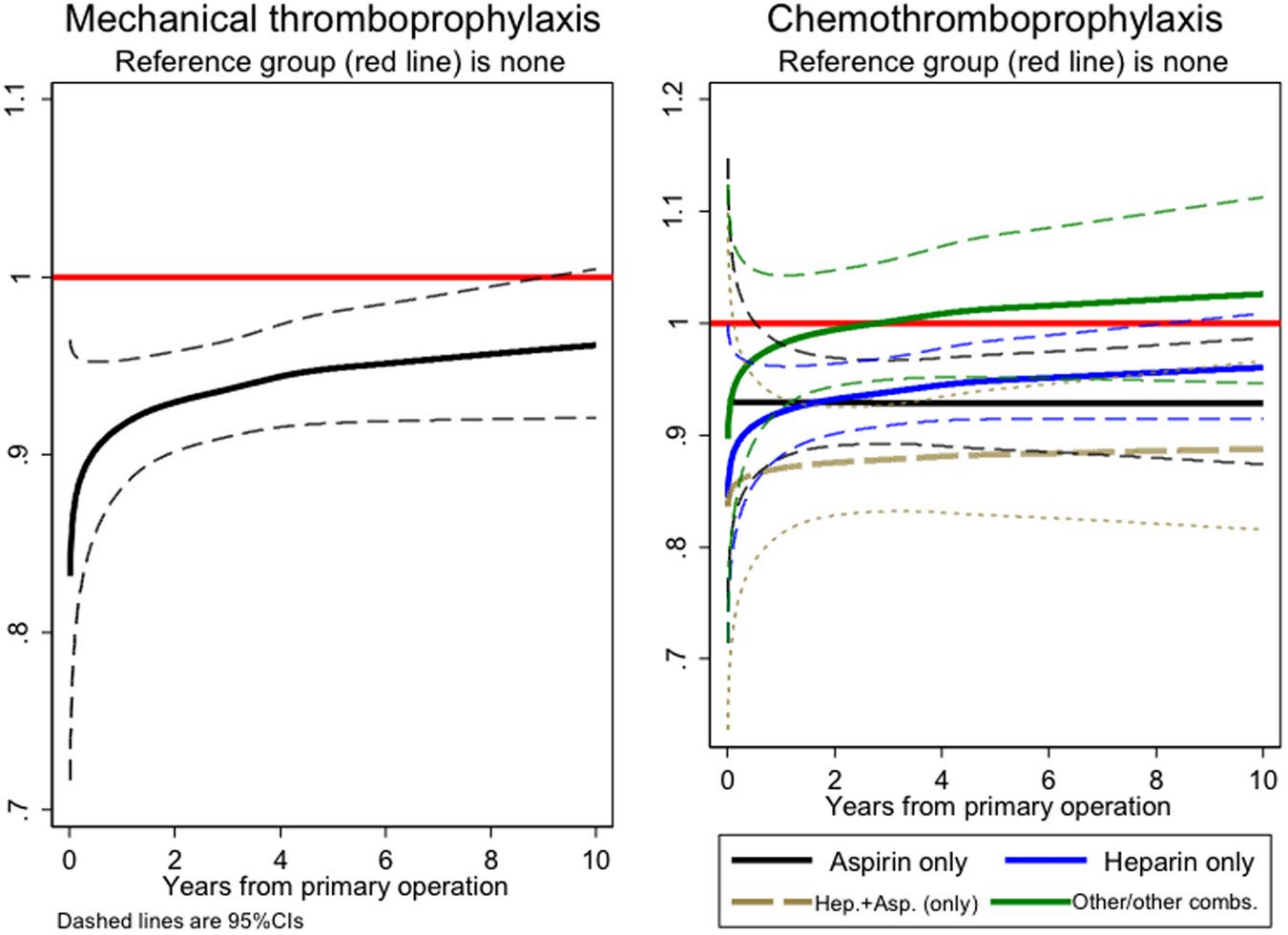
(**a**) and (**b**) Hazard rates ratios (with pointwise 95% CIs) associated with (**a**) mechanical and (**b**) chemical thromboprophylaxis for hips (adjusting for time varying effects of age, gender, year of operation, ASA, surgical approach, anaesthetic used and implant type).

Internal modelling showed a relative decrease in risk of death initially when mechanical thromboprophylaxis was used, this effect diminished with time (p = 0.01) over a long period, which was more suggestive of selection rather than causal effect (Fig. 2a). The external comparison found evidence of a group effect for mechanical thromboprophylaxis (p < 0.001); this was most marked in the perioperative period but those that received it had lower mortality at all time periods, though the time interaction was consistent with chance. There was a weak suggestion that those that received it had lower smoking-related mortality, which was more marked for respiratory system mortality (p = 0.07) (Table 2).

**Table 2:** Comparison of SMRs between those given or not given any mechanical thromboprophylaxis (i.e. ‘Yes’ vs. ‘No’) for hips. (Ratios of observed (O) to expected (E) numbers of deaths obtained from national rates [with 95% CI shown in parentheses]).

HIP PRIMARIES	Main cause of death (ICD10)					
	(i) All causes of death		(ii) Respiratory system (all ‘J’ codes)		(iii) ‘Cancers related to smoking’*	
	Yes (n = 366,820)	No** (n = 56,157)	Yes (n = 285,305)	No*** (n = 46,468)	Yes (n = 285,305)	No*** (n = 46,468)
	O/E	O/E	O/E	O/E	O/E	O/E
All deaths	26,939/46,694.3 = 0.58 [0.57–0.58]	6,566/10,038.2 = 0.65 [0.64–0.67]	2,287/5,301.2 = 0.43 [0.41–0.45]	615/1,210.2 = 0.51 [0.47–0.55]	3,701/5,062.2 = 0.73 [0.71–0.76]	898/1,148.0 = 0.78 [0.73–0.84]
**Deaths by time interval post primary:**						
Within 90 d	1,542/2,493.0 = 0.62 [0.59–0.65]	304/383.0 = 0.79 [0.71–0.89]	105/282.3 = 0.37 [0.30–0.45]	17/45.1 = 0.38 [0.22–0.60]	65/304.6 = 0.21 [0.16–0.27]	6/49.1 = 0.12 [0.04–0.27]
90 d to 1 y	2,939/7,320.3 = 0.40 [0.39–0.42]	545/1,177.0 = 0.46 [0.42–0.50]	216/834.4 = 0.26 [0.23–0.30]	31/139.6 = 0.22 [0.15–0.32]	479/878.7 = 0.55 [0.50–0.60]	82/148.4 = 0.55 [0.44–0.69]
1 y to 3 y	8,468/16,707.7 = 0.51 [0.50–0.52]	1,705/3,117.1 = 0.55 [0.52–0.57]	677/1,911.4 = 0.35 [0.33–0.38]	128/375.4 = 0.34 [0.28–0.41]	1,367/1,883.0 = 0.73 [0.69–0.77]	269/373.8 = 0.72 [0.64–0.81]
3 y to 5 y	7,587/11,683.6 = 0.65 [0.63–0.66]	1,896/2,745.9 = 0.69 [0.66–0.72]	686/1,326.6 = 0.52 [0.48–0.56]	204/331.2 = 0.62 [0.53–0.71]	1,061/1,204.0 = 0.88 [0.83–0.94]	285/306.7 = 0.93 [0.82–1.04]
5 y to 7 y	4,597/6,291.2 = 0.73 [0.71–0.75]	1,385/1,808.8 = 0.77 [0.73–0.81]	415/701.4 = 0.59 [0.54–0.65]	156/218.2 = 0.71 [0.61–0.84]	543/595.7 = 0.91 [0.84–0.99]	187/189.3 = 0.99 [0.85–1.14]
7 y+	1,806/2,198.4 = 0.82 [0.78–0.86]	732/806.2 = 0.91 [0.84–0.98]	188/245.1 = 0.77 [0.66–0.89]	79/100.6 = 0.78 [0.62–0.98]	186/196.1 = 0.95 [0.82–1.09]	69/80.8 = 0.85 [0.66–1.08]
Post 90-day modelling: Group x period interaction; Group difference****	**Yes vs. No:** P = 0.255; P < 0.001		**Yes vs. No:** P = 0.352; P = 0.069		**Yes vs. No:** P = 0.650; P = 0.585	

*See text for details.

**Excludes 1,179 cases where thromboprophylaxis was missing. ***Excludes 961 cases where thromboprophylaxis was missing.

****Assessed in the absence of a significant group x period interaction (but assuming a common slope).

### Chemical thromboprophylaxis

In the internal comparison, compared with a referent group of ‘no chemoprophylaxis’ (red), ‘aspirin only’ (black) was associated with a reduction in mortality that persisted (Fig. 2b). ‘Heparin + aspirin’ (brown dashed), was associated with an initial marked reduction that slowly receded; ‘Heparin only’ (blue), was associated with a less marked reduction that also slowly receded; a similar but again less marked effect was seem with ‘other chemoprophylaxis/other combinations’ (green).

For the external comparison, chemical thromboprophylaxis was grouped into aspirin alone, other combinations and none. The external comparison showed slightly different results, both aspirin and other combinations had lower 90-day mortality compared to none but after this period, all-cause mortality was slightly lower for aspirin (group effect p = 0.009) than those with none, with no evidence of a time interaction (Table 3). Similarly, there was little difference in smoking related cancer mortality for any of the groups suggesting little or no selection. Interestingly, there was a persistent reduction in circulatory disease deaths as aspirin had a lower SMR for all time periods than no aspirin (data not shown; group difference p = 0.007) which would be consistent with a longer term cardioprotective effect.

**Table 3:** Comparison of SMRs between subgroups of chemical thromboprophylaxis for hips (‘aspirin only’, ‘others/other combinations’ (see table footnote) and ‘none’). (Ratios of observed (O) to expected (E) numbers of deaths obtained from national rates [with 95% CI shown in parentheses]).

HIP PRIMARIES	Main cause of death (ICD10)											
	(i) All causes of death				(ii) Respiratory system (all ‘J’ codes)				(iii) ‘Cancers related to smoking’*			
	Aspirin only (n = 47,739)	Others/other combinations (n = 328,580)		None** (n = 46,658)	Aspirin only (n = 34,297)	Others/other combinations (n = 265,258)		None*** (n = 32,218)	Aspirin only (n = 34,297)	Others/other combinations (n = 265,258)		None*** (n = 32,218)
	O/E	O/E		O/E	O/E	O/E		O/E	O/E	O/E		O/E
All deaths	4,871/8,241.2 = 0.59 [0.57–0.61]	23,273/39,932.4 = 0.58 [0.58–0.59]		5,361/8,558.8 = 0.63 [0.61–0.64]	443/901.6 = 0.49 [0.45–0.54]	2,009/4,720.3 = 0.43 [0.41–0.44]		450/889.4 = 0.51 [0.46–0.55]	645/846.4 = 0.76 [0.70–0.82]	3,305/4,535.4 = 0.73 [0.70–0.75]		649/828.4 = 0.78 [0.72–0.85]
**Deaths by time interval post primary:**												
Within 90d	218/323.7 = 0.67 [0.59–0.77]	1,380/2,226.9 = 0.62 [0.59–0.65]		248/325.4 = 0.76 [0.67–0.86]	19/34.2 = 0.56 [0.33–0.87]	87/260.6 = 0.33 [0.27–0.41]		16/32.6 = 0.49 [0.28–0.80]	9/36.8 = 0.24 [0.11–0.46]	50/282.4 = 0.18 [0.13–0.23]		12/34.5 = 0.35 [0.18–0.61]
90 d to 1 y	411/998.1 = 0.41 [0.37–0.45]	2,648/6,491.9 = 0.41 [0.39–0.42]		425/1,007.4 = 0.42 [0.38–0.46]	29/106.2 = 0.27 [0.18–0.39]	190/766.0 = 0.25 [0.21–0.29]		28/101.8 = 0.28 [0.18–0.40]	68/111.7 = 0.61 [0.47–0.77]	438/809.9 = 0.54 [0.49–0.59]		55/105.5 = 0.52 [0.39–0.68]
1 y to 3 y	1,342/2,641.6 = 0.51 [0.48–0.54]	7,423/14,480.18 = 0.51 [0.50–0.52]		1,408/2,703.1 = 0.52 [0.49–0.55]	108/287.0 = 0.38 [0.31–0.45]	605/1,722.1 = 0.35 [0.32–0.38]		92/277.7 = 0.33 [0.27–0.41]	206/283.3 = 0.73 [0.63–0.83]	1,232/1,703.7 = 0.72 [0.68–0.76]		198/269.8 = 0.73 [0.64–0.84]
3 y to 5 y	1,377/2,245.2 = 0.61 [0.58–0.65]	6,498/9,800.9 = 0.66 [0.65–0.68]		1,607/2,383.4 = 0.67 [0.64–0.71]	139/247.2 = 0.56 [0.47–0.66]	601/1,160.7 = 0.52 [0.48–0.56]		150/250.0 = 0.60 [0.51–0.70]	195/225.7 = 0.86 [0.75–0.99]	937/1,058.1 = 0.89 [0.83–0.94]		214/226.8 = 0.94 [0.82–1.08]
5 y to 7 y	1,048/1,445.7 = 0.72 [0.68–0.77]	3,780/5,105.0 = 0.74 [0.72–0.76]		1,154/1,549.3 = 0.74 [0.70–0.79]	94/160.3 = 0.59 [0.47–0.72]	373/595.6 = 0.63 [0.56–0.69]		104/163.8 = 0.63 [0.52–0.77]	127/136.1 = 0.93 [0.78–1.11]	480/508.9 = 0.94 [0.86–1.03]		123/140.0 = 0.88 [0.73–1.05]
7 y+	475/587.0 = 0.81 [0.74–0.89]	1,544/1,827.4 = 0.84 [0.80–0.89]		519/590.3 = 0.88 [0.81–0.96]	54/66.7 = 0.81 [0.61–1.06]	153/215.4 = 0.71 [0.60–0.83]		60/63.6 = 0.94 [0.72–1.22]	40/52.7 = 0.76 [0.54–1.03]	168/172.4 = 0.97 [0.83–1.13]		47/51.8 = 0.91 [0.67–1.21]
Post 90-day modelling: Group x period interaction; Group diff.****	**Aspirin vs None:** P = 0.569; P = 0.009		**Other vs None:** P = 0.561; P = 0.241		**Aspirin vs None:** P = 0.290; P = 0.637		**Other vs None:** P = 0.459; P = 0.136		**Aspirin vs None:** P = 0.513; P = 0.748		**Other vs None:** P = 0.384; P = 0.948	

*See text for details.

**Excludes 1,179 cases where thromboprophylaxis was missing. ***Excludes 961 cases where thromboprophylaxis was missing.

****Group difference assessed in the absence of a significant group x period interaction (but assuming a common slope);

‘Others/other combinations’ here includes Heparin, Heparin + aspirin plus other thromboprophylaxis agents plus any combinations of these, either with each other or with aspirin and/or Heparin.

### Type of anaesthesia

**Figure 3:**
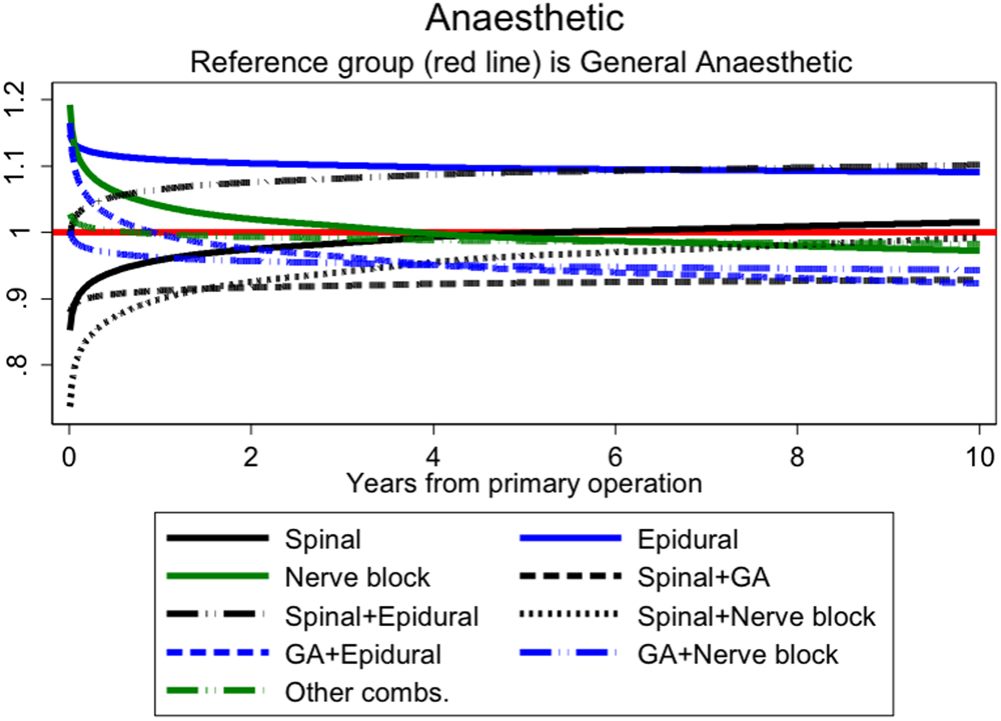
Hazard rates ratios associated with anaesthetic for hips (adjusting for time varying effects of age, gender, year of operation, ASA, surgical approach, mechanical prophylaxis, chemical prophylaxis and implant type). The referent group is ‘GA only’ (red line); other groups are as shown in the legend but 95% CIs have been omitted for simplification.

The internal comparison found that compared with general anaesthetic (GA), spinal anaesthetic was more advantageous early on but this effect diminished with time, whilst spinal plus GA seemed to show persistent reduced mortality (Fig. 3). For the external comparison, anaesthesia was grouped into spinal only, GA only, spinal plus GA and other combinations. The most marked difference was for spinal plus GA versus GA where the former group had lower mortality at all time points (Table 4). This was also seen for smoking related cancers and there was evidence of a group by time interaction (p = 0.03) consistent with selection. Spinal alone compared to GA was more suggestive of a causal effect as the mortality benefits were only seen in the perioperative period and there was no suggestion of reduced smoking-related cancer mortality. Respiratory deaths were, if anything, higher in this group compared to the GA group suggesting that there may be more, rather than less, comorbidity.

**Table 4:** Comparison of SMRs between specific anaesthetic groups for hips (‘Spinal only’, ‘GA only’, ‘Spinal + GA only’ and ‘others/other combination’ (see table footnote). (Ratios of observed (O) to expected (E) numbers of deaths obtained from national rates [with 95% CI shown in parentheses]).

HIP PRIMARIES	‘Main’ cause of death (ICD10)								
	(i) All causes of death				(ii) Respiratory system (all ‘J’ codes)				
	Spinal only (n = 177,112)	GA only (n = 100,218)	Spinal + GA only (n = 51,747)	Others/Other combinations** (n = 83,461)	Spinal only (n = 143,511)	GA only (n = 73,636)		Spinal + GA only (n = 39,409)	Others/Other combinations*** (n = 66,322)
	O/E	O/E	O/E	O/E	O/E	O/E		O/E	O/E
All deaths	13,035/22,272.3 = 0.59 [0.58–0.60]	7,763/13,029.4 = 0.60 [0.58–0.61]	3,457/6,562.5 = 0.53 [0.51–0.54]	7,945/12,851.3 = 0.62 [0.60–0.63]	1,197/2,635.0 = 0.45 [0.43–0.48]	560/1,393.8 = 0.40 [0.37–0.44]		242/709.9 = 0.34 [0.30–0.39]	771/1,522.4 = 0.51 [0.47–0.54]
**Deaths by time interval post primary:**									
Within 90 d	772/1,242.9 = 0.62 [0.58–0.67]	445/642.1 = 0.69 [0.63–0.76]	178/338.9 = 0.53 [0.45–0.61]	398/575.2 = 0.69 [0.63–0.76]	49/146.5 = 0.33 [0.25–0.44]	27/68.2 = 0.40 [0.26–0.58]	9/36.8 = 0.24 [0.11–0.46]		30/66.6 = 0.45 [0.30–0.64]
90 d to 1 y	1,458/3,621.1 = 0.40 [0.38–0.42]	758/1,899.6 = 0.40 [0.37–0.43]	375/999.6 = 0.38 [0.34–0.42]	784/1,734.7 = 0.45 [0.42–0.48]	103/429.8 = 0.24 [0.20–0.29]	47/203.1 = 0.23 [0.17–0.31]	18/109.3 = 0.16 [0.10–0.26]		63/202.1 = 0.31 [0.24–0.40]
1 y to 3 y	4,087/8,079.9 = 0.51 [0.49–0.52]	2,335/4,488.2 = 0.52 [0.50–0.54]	1,062/2,292.0 = 0.46 [0.44–0.69]	2,316/4,282.2 = 0.54 [0.52–0.56]	363/962.5 = 0.38 [0.34–0.42]	141/484.0 = 0.29 [0.25–0.34]	71/250.4 = 0.28 [0.22–0.36]		181/505.0 = 0.36 [0.31–0.41]
3 y to 5 y	3,703/5,487.6 = 0.67 [0.65–0.70]	2,113/3,253.4 = 0.65 [0.62–0.68]	950/1,628.8 = 0.58 [0.55–0.62]	2,234/3,398.4 = 0.66 [0.63–0.69]	382/647.0 = 0.59 [0.53–0.65]	165/348.4 = 0.47 [0.40–0.55]	67/174.7 = 0.38 [0.30–0.49]		232/404.9 = 0.57 [0.50–0.65]
5 y to 7 y	2,161/2,885.0 = 0.75 [0.72–0.78]	1,420/1,908.1 = 0.74 [0.71–0.78]	605/929.6 = 0.65 [0.60–0.70]	1,520/2,030.2 = 0.75 [0.71–0.79]	207/334.7 = 0.62 [0.54–0.71]	113/201.0 = 0.56 [0.46–0.68]	50/98.9 = 0.51 [0.38–0.67]		179/242.2 = 0.74 [0.63–0.86]
7 y+	854/955.8 = 0.89 [0.83–0.96]	692/837.9 = 0.83 [0.77–0.89]	287/373.5 = 0.77 [0.68–0.86]	693/830.5 = 0.83 [0.77–0.90]	93/114.4 = 0.81 [0.66–1.00]	57/89.2 = 0.64 [0.48–0.83]	27/39.9 = 0.68 [0.45–0.98]		86/101.6 = 0.85 [0.68–1.05]
Post 90-day modelling: Group x period interaction; Group difference ****	**Spinal vs. GA:** P = 0.071; P = 0.386		**Spinal + GA vs. GA:** P = 0.874; P < 0.001		**Spinal vs. GA:** P = 0.615; P < 0.001		**Spinal + GA vs. GA:** P = 0.417; P = 0.081		
**HIP PRIMARIES**	**‘Main’ cause of death (ICD10)**								
	(iii) ‘Cancers related to smoking’*								
	**Spinal only (n = 143,511)**		**GA only (n = 73,636)**		**Spinal + GA only (n = 39,409)**			Others/Other combinations*** (n = 66,322)	
	**O/E**		**O/E**		**O/E**			**O/E**	
All deaths	1,881/2,507.1 = 0.75 [0.72–0.78]		1,010/1,353.3 = 0.75 [0.70–0.79]		467/684.0 = 0.68 [0.62–0.75]			1,048/1,434.4 = 0.73 [0.69–0.78]	
**Deaths by time interval post primary:**									
With in 90 d	29/157.5 = 0.18 [0.12–0.26]		17/75.1 = 0.23 [0.13–0.36]		10/40.3 = 0.25 [0.12–0.46]			13/71.2 = 0.18 [0.10–0.31]	
90 d to 1 y	259/450.5 = 0.57 [0.51–0.65]		106/218.3 = 0.49 [0.40–0.59]		65/116.9 = 0.56 [0.43–0.71]			118/211.6 = 0.56 [0.46–0.67]	
1 y to 3 y	679/944.3 = 0.72 [0.67–0.78]		356/486.1 = 0.73 [0.66–0.81]		172/249.3 = 0.69 [0.59–0.80]			358/495.9 = 0.72 [0.65–0.80]	
3 y to 5 y	548/584.8 = 0.94 [0.86–1.02]		280/324.2 = 0.86 [0.77–0.97]		141/159.9 = 0.88 [0.74–1.04]			303/367.2 = 0.83 [0.73–0.92]	
5 y to 7 y	275/280.6 = 0.98 [0.87–1.10]		180/176.3 = 1.02 [0.88–1.18]		55/85.1 = 0.65 [0.49–0.84]			184/206.8 = 0.89 [0.77–1.03]	
7 y+	91/89.4 = 1.02 [0.82–1.25]		71/73.4 = 0.97 [0.76–1.22]		24/32.5 = 0.74 [0.47–1.10]			72/81.5 = 0.88 [0.69–1.11]	
Post 90-day modelling: Group x period interaction; Group difference ****	**Spinal vs. GA:** P = 0.806; P = 0.350				**Spinal + GA vs. GA:** P = 0.034; N/A				

*See text for details.

**Excludes 11,618 cases where anaesthetic was missing. ***Excludes 9,856 cases where anaesthetic was missing.

****Assessed in the absence of a significant group x period interaction (but assuming a common slope).

### Type of hip prosthesis

**Figure 4:**
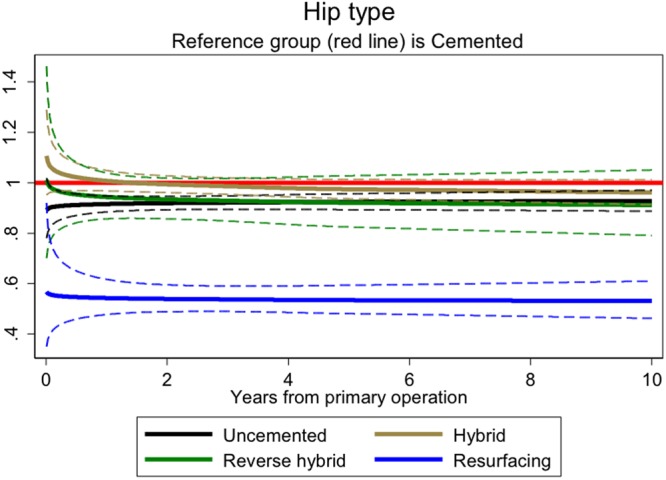
Hazard rates ratios associated with hip implant type (adjusting for time varying effects of age, gender, year of operation, ASA, surgical approach, anaesthetic used, mechanical prophylaxis and chemical prophylaxis).

The internal modelling found that the resurfacing hazard ratios remained lower than the cemented referent group over time (Fig. 4). A similar time-invariant pattern was seen with uncemented hips. In the external comparison, resurfacing was associated with reduced all cause (p-value for interaction = 0.01) and smoking-related cancer mortality that was seen over the follow-up period consistent with a selection effect (Table 5).

**Table 5:** Comparison of SMRs between hip resurfacings, uncemented total hip replacements and other types of hip implants (i.e. cemented, hybrid and reverse hybrid). (Ratios of observed (O) to expected (E) numbers of deaths obtained from national rates [with 95% CI shown in parentheses]).

HIP PRIMARIES	Main cause of death (ICD10)											
	(i) All causes of death				(ii) Respiratory system (all ‘J’ codes)				(iii) ‘Cancers related to smoking’*			
	Resurfacings (n = 27,921)	Uncemented (n = 158,874)		Other hip types** (n = 237,330)	Resurfacings (n = 17,557)	Uncemented (n = 124,338)		Other hip types*** (n = 190,816)	Resurfacings (n = 17,557)	Uncemented (n = 124,338)		Other hip types*** (n = 190,816)
	O/E	O/E		O/E	O/E	O/E		O/E	O/E	O/E		O/E
All deaths	569/1,277.6 = 0.45 [0.41–0.48]	7823/13,591.3 = 0.58 [0.56–0.59]		25,361/42,241.3 = 0.60 [0.59–0.61]	12/73.2 = 0.16 [0.08–0.29]	636/1,466.0 = 0.43 [0.40–0.47]		2,280/5,016.9 = 0.45 [0.44–0.47]	87/178.7 = 0.49 [0.39–0.60]	1,265/1,725.1 = 0.73 [0.69–0.77]		3,276/4,347.9 = 0.75 [0.73–0.78]
**Deaths by time interval post primary:**												
Within 90 d	26/46.6 = 0.56 [0.36–0.82]	462/789.5 = 0.59 [0.53–0.64]		1,361/2,048.5 = 0.67 [0.63–0.70]	0/2.3 = 0.0 [0.0–1.59]	29/83.1 0.35 [0.23–0.50]		93/242.9 = 0.38 [0.31–0.47]	1/6.3 = 0.16 [0.00–0.89]	16/109.1 = 0.15 [0.08–0.24]		54/239.3 = 0.23 [0.17–0.29]
90 d to 1 y	45/146.1 = 0.31 [0.22–0.41]	963/2,313.8 = 0.42 [0.38–0.44]		2,483/6,063.8 = 0.41 [0.39–0.43]	0/7.4 = 0.0 [0.0–0.50]	59/246.5 = 0.24 [0.18–0.31]		189/723.0 = 0.26 [0.23–0.30]	4/19.9 = 0.20 [0.05–0.52]	180/315.8 = 0.57 [0.49–0.66]		378/694.7 = 0.54 [0.49–0.60]
1 y to 3 y	172/399.0 = 0.43 [0.37–0.50]	2,697/5,159.4 = 0.52 [0.50–0.54]		7,338/14,342.8 = 0.51 [0.50–0.52]	2/21.7 = 0.09 [0.01–0.33]	213/558.1 = 0.38 [0.33–0.44]		592/1,715.8 = 0.35 [0.32–0.37]	23/55.8 = 0.41 [0.26–0.62]	485/670.8 = 0.72 [0.66–0.79]		1,134/1,539.0 = 0.75 [0.69–0.78]
3 y to 5 y	169/361.0 = 0.47 [0.40–0.54]	2,176/3,291.1 = 0.66 [0.63–0.69]		7,185/10,859.8 = 0.66 [0.65–0.68]	7/21.4 = 0.33 [0.13–0.67]	193/356.1 = 0.54 [0.47–0.62]		695/1,290.0 = 0.54 [0.50–0.58]	30/51.7 = 0.58 [0.39–0.83]	342/399.0 = 0.86 [0.77–0.95]		978/1,069.2 = 0.91 [0.86–0.97]
5 y to 7 y	108/231.8 = 0.47 [0.38–0.56]	1,129/1,560.0 = 0.72 [0.68–0.77]		4,805/6,394.3 = 0.75 [0.73–0.77]	1/14.5 = 0.07 [0.00–0.38]	104/168.6 = 0.62 [0.50–0.75]		475/746.8 = 0.64 [0.58–0.70]	20/32.9 = 0.61 [0.37–0.94]	183/177.6 = 1.03 [0.89–1.19]		535/583.7 = 0.92 [0.84–1.00]
7y+	49/93.1 = 0.53 [0.39–0.70]	396/477.6 = 0.83 [0.75–0.92]		2,187/2,532.1 = 0.86 [0.83–0.90]	2/5.8 = 0.34 [0.04–1.24]	38/53.6 = 0.71 [0.50–0.97]		236/298.4 = 0.79 [0.69–0.90]	9/12.2 = 0.74 [0.34–1.40]	59/52.7 = 1.12 [0.85–1.44]		197/222.1 = 0.89 [0.77–1.02]
Post 90-day modelling: Group x period Interaction; Group difference****	**Resurfacing vs other:** P = 0.012; N/A		**Uncemented vs other:** P = 0.328; P = 0.905		**Resurfacing vs other:** P = 0.911; P < 0.001		**Uncemented vs other:** P = 0.686; P = 0.764		**Resurfacing vs other:** P = 0.252; P < 0.001		**Uncemented vs other:** P = 0.140; P = 0.731	

*See text for details.

**Excludes 31 where hip type was uncertain. ***Excludes 23 where hip type was uncertain.

****Assessed in the absence of a significant group x period interaction (but assuming a common slope).

Supplementary Material Fig. S4 shows results from the internal model which take into account bearing surface. Supplementary Material Fig. S5 shows results for grouped BMI.

### Type of knee prosthesis

**Figure 5:**
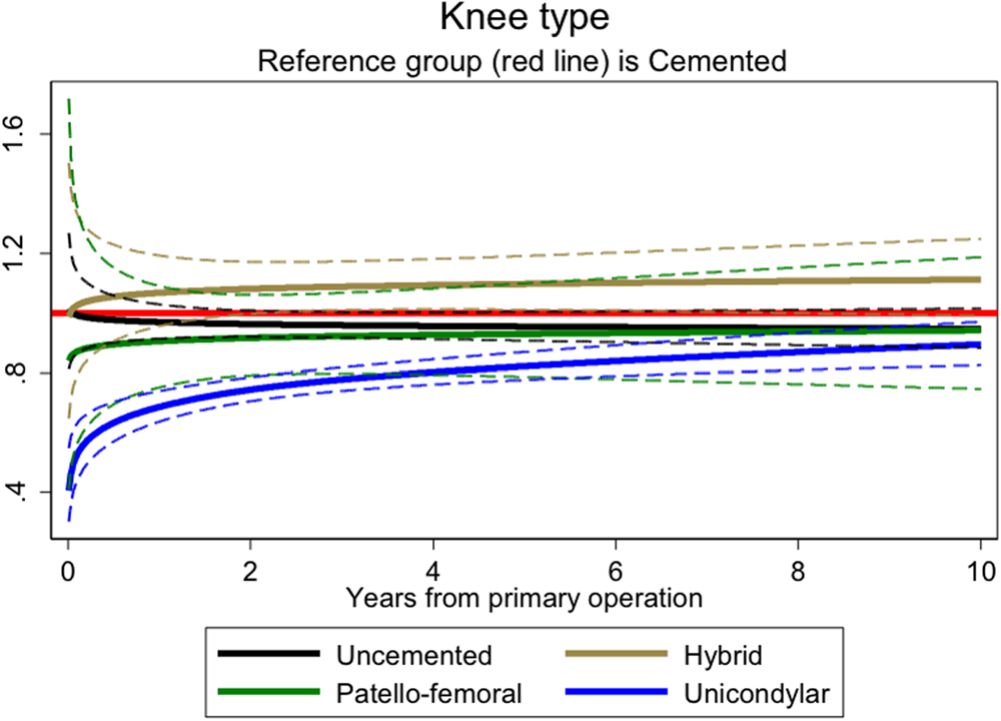
Hazard rates ratios for mortality after knee replacement associated with type of knee (adjusting for time varying effects of age, gender, year group and ASA; n = 469,952). The referent group ‘cemented’ is shown as a red line; solid black, brown, green and blue denote ‘uncemented, ‘hybrid’, ‘patellofemoral’ and ‘unicondylar’ respectively; 95% CIs shown as dashed lines in the same colours.

The internal comparison showed a survival benefit with the use of unicondylar knee replacements that waned over time (Fig. 5). The external comparison found a group effect (p < 0.001) though all-cause mortality was reduced across all the time periods and was not restricted to the early period. There was no evidence of reduced smoking-related cancers but respiratory deaths were reduced in the intervention group (SMR 0.30, 95% CI 0.24, 0.36 versus 0.38, 95% CI 0.36, 0.39, p = 0.01) (Table 6).

**Table 6:** Comparison of SMRs between unicondylar knees and other knee implant types. (Ratios of observed (O) to expected (E) numbers of deaths obtained from national rates [with 95% CI shown in parenthesis]).

KNEE PRIMARIES	Main cause of death (ICD10)					
	(i) All causes of death		(ii) Respiratory system (all ‘J’ codes)		(iii) ‘Cancers related to smoking’*	
	Unicondylar only (n = 41,619)	All other knee types** (n = 428,333)	Unicondylar only (n = 30,661)	All other knee types*** (n = 353,600)	Unicondylar only (n = 30,661)	All other knee types*** (n = 353,600)
	O/E	O/E	O/E	O/E	O/E	O/E
All deaths	1,649/36,378.0 = 0.45 [0.43–0.48]	34,346/60,987.6 = 0.56 [0.56–0.57]	108/364.7 = 0.30 [0.24–0.36]	2,824/7,507.4 = 0.38 [0.36–0.39]	281/469.8 = 0.60 [0.53–0.67]	4,714/7,182.7 = 0.66 [0.64–0.68]
**Deaths by time interval post primary:**						
Within 90 d	45/174.0 = 0.26 [0.19–0.35]	1,676/3,044.8 = 0.55 [0.52–0.58]	1/16.2 = 0.06 [0.00–0.34]	110/362.8 = 0.30 [0.25–0.37]	0/24.4 = 0.00 [0.00–0.15]	38/405.6 = 0.09 [0.07–0.13]
90 d to 1 y	131/522.7 = 0.25 [0.21–0.30]	3,228/9,042.5 = 0.36 [0.34–0.37]	5/49.4 = 0.10 [0.03–0.24]	194/1,088.5 = 0.18 [0.15–0.21]	28/72.3 = 0.39 [0.26–0.56]	478/1,182.4 = 0.40 [0.37–0.44]
1 y to 3 y	490/1,247.7 = 0.39 [0.36–0.43]	10,465/21,273.7 = 0.49 [0.48–0.50]	25/121.5 = 0.21 [0.13–0.30]	755/2,602.3 = 0.29 [0.27–0.31]	79/166.4 = 0.47 [0.38–0.59]	1,792/2,614.7 = 0.69 [0.65–0.72]
3 y to 5 y	502/941.8 = 0.53 [0.49–0.58]	9,845/15,614.8 = 0.63 [0.62–0.64]	34/96.7 = 0.35 [0.24–0.49]	872/1,936.4 = 0.45 [0.42–0.48]	100/119.4 = 0.84 [0.68–1.02]	1,388/1,756.5 = 0.79 [0.75–0.83]
5 y to 7 y	347/544.3 = 0.64 [0.57–0.71]	6,431/8,734.7 = 0.74 [0.72–0.75]	30/57.9 = 0.52 [0.35–0.74]	593/1,091.0 = 0.54 [0.50–0.59]	54/64.4 = 0.84 [0.63–1.09]	760/902.3 = 0.84 [0.78–0.90]
7 y+	134/207.5 = 0.65 [0.54–0.76]	2,701/3,277.0 = 0.82 [0.79–0.86]	13/22.9 = 0.57 [0.30–0.97]	300/426.4 = 0.70 [0.63–0.79]	20/22.9 = 0.87 [0.53–1.35]	258/321.1 = 0.80 [0.71–0.91]
Post 90-day modelling: Group x period interaction; Group difference ****	**Unicondylar vs other:** P = 0.203; P < 0.001		**Unicondylar vs other:** P = 0.329; P = 0.014		**Unicondylar vs other:** P = 0.110; P = 0.102	

*See text for details.

**Excludes 37 where knee type was uncertain. ***Excludes 30 where knee type was uncertain.

****Assessed in the absence of a significant group x period interaction (but assuming a common slope).

## Discussion

We have used the extended mortality follow-up from the NJR to explore where some of the previously reported “protective” surgical-related exposures are truly causally related or the result of confounding. The reduced mortality seen for patients undergoing joint replacement was markedly attenuated, though not totally abolished, over time, highlighting a healthy selection effect, a finding consistent with previous studies^17–21^.

Of seven interventions studied, we believe three interventions (hip resurfacing, combined spinal and general anaesthetic, unicondylar knee implants) showed patterns more consistent with confounding by indication; healthier patients being more likely to have received the intervention. The evidence for this seemed strongest for hip resurfacing where patients showed persistent reduced mortality from all cause and smoking-related cancers. This type of hip replacement has specifically been marketed for the more active patient, reflected in the demographics observed^6^. Possibly earlier/better mobilisation might contribute to the decreased mortality^22^, however, this is unlikely to explain the observed difference in smoking-related deaths. The results for the combined spinal and GA anaesthesia group were similar, the patients had better all-cause mortality and a time interaction for smoking–related cancers. Similar results were seen for unicondylar knee replacement; although there no difference for smoking-related cancers, the reduction in respiratory deaths suggested a selection effect.

Mechanical thromboprophylaxis was less consistent with an apparent genuine intervention-related reduction. There was little evidence that those receiving intervention were otherwise healthier and the internal comparison found a slow waning effect of the mortality risk over time, so we remain unsure. Chemoprophylaxis with aspirin was interesting as aspirin was associated with a reduction in all-cause mortality which was sustained over time and this was mirrored in fewer circulatory disease deaths. We have assumed that any causal perioperative intervention would only exert its effect in the early postoperative period. However, it is possible that previously untreated patients with heart disease who are given aspirin perioperatively keep taking it, leading to decreased risk of mortality from cardiac events^23,24^. The mortality patterns for a posterior approach were ambiguous, suggesting a selection effect but also possibly a causal benefit with only short-term mortality gains. There was no evidence of lower smoking related mortality but weak evidence of reduced respiratory deaths in the posterior approach group. This may be because the posterior approach was associated with less bleeding, tissue damage^25^ and better early mobilisation^26^, hence reduced risk of complications such as thrombosis and therefore a reduction in early deaths. In the longer term, preservation of the abductor muscles in the posterior group compared to the most common alternative (lateral/anterolateral approach) may lead to improved mobility^27^ and a slow attenuation and protection from respiratory deaths. There is unlikely to be a selection effect as the vast majority of hip replacements are performed by surgeons who use either the posterior or lateral/anterolateral approach with no patient level selection. Some selection is observed amongst surgeons who perform minimally invasive or anterior approaches for patients with lower body mass index but such procedures account for only 4% of the cases recorded by the NJR^28^. Most convincing were the results for those receiving spinal anaesthetic. Here only perioperative mortality was reduced and if anything, respiratory mortality was higher in the spinal group suggesting that patients with worse lung function may have been selected for this type of anaesthesia. Spinal anaesthesia has been demonstrated to be associated with lower risk of complications such as surgical site infection, pulmonary complication, blood transfusion, thromboembolic events, prolonged length of stay and intensive care unit admission when compared to general anaesthesia^29^. Avoidance of these complications may therefore reduce the risk of mortality. Smaller evidence synthesis studies have only demonstrated a reduced length of stay in spinal compared to general anaesthesia^30^ but this may reflect the small overall sample size and the need for follow up beyond hospital discharge to assess the effect of the type of anaesthesia on mortality; as recommended by Johnson *et al*.^30^, our analysis includes consideration of intermediate and long-term outcome.

The NJR is the largest joint replacement register in the world^6^. Linkage with other comprehensive national databases allows good data coverage but the data are observational and causality cannot usually be proven. However, we feel that triangulating findings across different analytical strategies points more strongly to causation and may be an analytical strategy for trying to infer causality with other large sets of observational data when it is not feasible to undertake randomised controlled trials.

Data collection occurs at the point of surgical treatment and as such, variables that may change post operatively, such as type of thromboprophylaxis cannot be reliably captured. Whilst the NJR does have a range of variables that attempt to adjust for case-mix/comorbidity, residual confounding always remains a possibility due to lack of good indicators of disease severity or early stage conditions, such as mild cognitive impairment or early dementia. Replication of our results in other national registries would be helpful especially if the patient selection factors differ from the UK, for example in predominantly private health care systems.

We present further data supporting the potential causal role for aspirin chemothromboprophylaxis, posterior approach and spinal anaesthetic in THR in decreasing post-operative mortality and we recommend that patients undergoing primary THR are treated with these modalities to reduce the risk of mortality. We believe that the apparent “protective” effect of hip resurfacing, spinal and GA, unicondylar knee replacement and mechanical thromboprophylaxis are more likely to be due to selection though the last two are more difficult to interpret and may have a combination of both selection and causal effects, requiring further investigation.

## Electronic supplementary material

Supplementary material

## Data Availability

The data that support the findings of this study are available from the National Joint Registry but restrictions apply to the availability of these data, which were used under license for the current study, and so are not publicly available.
