# Supplementary material for: Using long term mortality to determine which perioperative risk factors of mortality following hip and knee replacement may be causal
Source: Sci Rep. 2018 Oct 9;8:15026. doi: 10.1038/s41598-018-33314-0 (PMC6177450; doi:10.1038/s41598-018-33314-0)

Using long term mortality to determine which perioperative risk factors of mortality following hip and knee replacement may be causal

Authors: Linda P Hunt[1], Michael R Whitehouse[1+2], Peter W Howard[3], Yoav Ben-Shlomo[4*], Ashley W Blom[1+2*]

[*]These authors contributed equally to this work

Supplementary Material Table 1: (i) Prognostic variables for hip analysis (n=424,156); reference categories for hazard rate ratios are marked with asterisks.

Variable	Subgroups	Number (%) *unless stated*
**Gender**	Male	173,390 (40.9%)
	Female*	250,766 (59.1%)
**Age (years)**	*Mean=69 Median= 69 (Inter-quartile range 62-76)*	
**Year of primary**	2003-5*	70,022 (16.5%)
	2006-8	139,732 (32.9%)
	2009-12	214,402 (50.6%)
**ASA**	P1*	80,210 (18.9%)
	P2	286,517 (67.6%)
	P3	55,431 (13.1%)
	P4/P5	1,998 (0.5%)
**Surgical approach**	Posterior	218,566 (51.5%)
	Other*	205,590 (48.5%)
**Mechanical prophylaxis**	No*	56,157 (13.2%)
	Yes	366,820 (86.5%)
	Missing	*1,179 (0.3%)*
**Chemical prophylaxis**	None*	46,658 (11.0%)
	Aspirin only	47,739 (11.3%)
	Heparin only	247,049 (58.2%)
	Heparin + aspirin	25,056 (5.9%)
	Others/other combinations	56,475 (13.3%)
	Missing	*1,179 (0.3%)*
**Anaesthetic**	Spinal only	177,112 (41.8%)
	GA only*	100218 (23.6%)
	Epidural only	14,950 (3.5%)
	Nerve block only	3,118 (0.7%)
	Spinal + GA	51,747 (12.2%)
	Spinal + Epidural	6,192 (1.5%)
	Spinal + Nerve block	11,158 (2.6%)
	GA + Epidural	15,693 (3.7%)
	GA + Nerve Block	29,089 (6.9%)
	Other combinations	3,261 (0.8%)
	Missing	*11,618 (2.7%)*
**Hip type**	Cemented*	163,578 (38.6%)
	Uncemented	158,874 (37.5%)
	Hybrid	64,248 (15.2%)
	Reverse hybrid	9,504 (2.2%)
	Resurfacing	27,921 (6.6%)
	Missing	*31 (<0.1%)*
**BMI group**	Normal (19-25 kg/m^2)*	54,748 (12.9%)
	Underweight (<19 kg/m^2)	1,655 (0.4%)
	Overweight (26-30 kg/m^2)	76,153 (18.0%)
	Obese (>30 kg/m^2)	56,645 (13.4%)
	Missing	*234,955 (55.4%)*

Supplementary Material Table 1: (ii) Further division of hip types by bearing surface

Hip fixation	Bearing surface	Number (%)
**Cemented**	MoP	145,617 (34.3%)
	MoM	988 (0.2%)
	CoP	13,134 (3.1%)
	Other/unsure	*3,839 (0.9%)*
**Uncemented**	MoP	59,936 (14.1%)
	MoM	21,651 (5.1%)
	CoP	22,175 (5.2%)
	CoC	50,397 (11.9%)
	CoM	1,585 (0.4%)
	Other/unsure	*3,130 (0.7%)*
**Hybrid**	MoP	43,311 (10.2%)
	MoM	1,694 (0.4%)
	CoP	7,233 (1.7%)
	CoC	10,752 (2.5%)
	Other/unsure	*1,258 (0.3%)*
**Reverse hybrid**	MoP	6,500 (1.5%)
	CoP	2,945 (0.7%)
	Other/unsure	*59 (<0.01%)*
**Resurfacing**	MoM	27,921 (6.6%)
**Unknown**	*Unknown*	*31 (<0.1%)*

MoP = metal-on-polyethylene; MoM = metal-on-metal; CoP = ceramic-on-polyethylene; CoC = ceramic-on-ceramic; CoM = ceramic-on-metal

Supplementary Material Table 2: Prognostic variables for knee analysis (n=469,989); reference categories for hazard rate ratios are marked with asterisks.

**Variable**	**Subgroups**	**Number (%) *unless stated***
**Gender**	Male	203,373 (43.3%)
	Female*	266,616 (56.7%)
**Age (years)**	*Mean 69 Median 70 (Inter-quartile range 63-76)*	
**Year of primary**	2003-5*	71,265 (15.2%)
	2006-8	153,613 (32.7%)
	2009-12	245,111 (52.2%)
**ASA**	P1*	67,852 (14.4%)
	P2	334,128 (71.1%)
	P3	66,272 (14.1%)
	P4/P5	1,737 (0.4%)
**Knee type**	Cemented*	390,974 (83.2%)
	Uncemented	25,482 (5.4%)
	Hybrid	5,926 (1.3%)
	Patellofemoral	5,951 (1.3%)
	Unicompartmental	41,619 (8.9%)
	Missing	*37 (<0.1%)*
**BMI group**	Normal (19-25 kg/m^2)*	35,880 (7.6%)
	Underweight (<19 kg/m^2)	644 (0.1%)
	Overweight (26-30 kg/m^2)	82,472 (17.6%)
	Obese (>30 kg/m^2)	95,041 (20.2%)
	Missing	*255,952 (54.5%)*

Supplementary Material Text 1: Proportional Hazards

'Proportional hazards' models can be written on the natural logarithm of the cumulative hazards (ln(H)) scale as $\ln\{H(t/x_i)\} = \ln\{H_o(t)\} + x_i\beta$ where x_i are the covariates in the model and β is the vector of coefficients to be determined. In FPM, the log of the 'baseline' cumulative hazard ($\ln\{H_o(t)\}$) is modelled as a restricted cubic spline function of logarithmically-transformed time from the primary, ln(t). Time-varying effects of the model covariates can be explored by adding in terms which represent interactions between the covariates and the spline functions for the baseline hazard; splines with fewer knots (df) can be used for these than for the baseline [1]. The simplest time-dependent effects, with df=1, are linear relationships between the HRs and the log of the time from the operation, thus the hazard rate ratio decreases with time as a simple exponential decay.

1. Lambert PC, Royston P. Further development of flexible parametric models for survival analysis. SJ. 2009;9: 265–290.

Supplementary Material Text 2: Smoking Related Cancers

Trachea, Lung, Bronchus (C33-C34), Upper respiratory sites (C00-C14), Oesophagus (C15), Larynx (C32), Cervical (C53), Bladder (C67), Kidney and Renal Pelvis (C64-C66, C68), Stomach (C16), Pancreas (C25), Unspecified site (C80), Myeloid leukaemia (C92) [2].

1. Lifestyle Statistics, Health and Social Care Information Centre. Statistics on Smoking: England, 2013. 1st ed. digital.nhs.uk. London; 2013 p. 96.

Supplementary Material Figures 1:

Plots of the logged SMRs by time from primary operation, as calculated separately for the risk factor groups, and shown in Tables 1 to 6 in the main text
Note in a few instances where O=0 , for plotting purposes only, the SMR was arbitrarily calculated as 1/E (instead of O/E) for plotting purposes only.

SMRs from Table 1: Hip Posterior surgical approach (dashed line) vs other approaches (solid line)

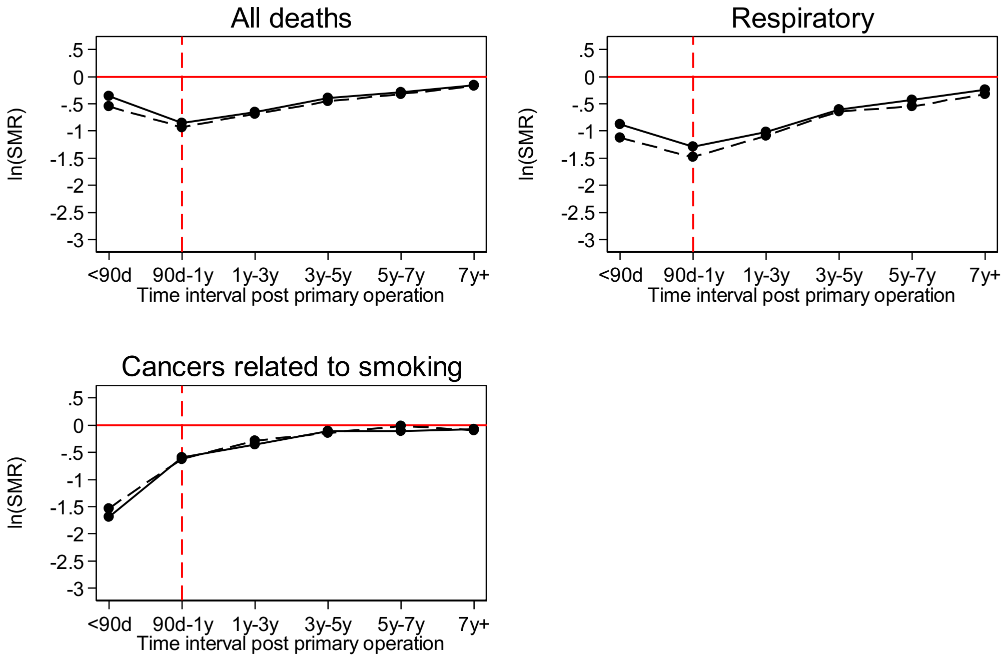

SMRs from Table 2: Hip Any Mechanical Prophylaxis (dashed line) vs None (solid line)

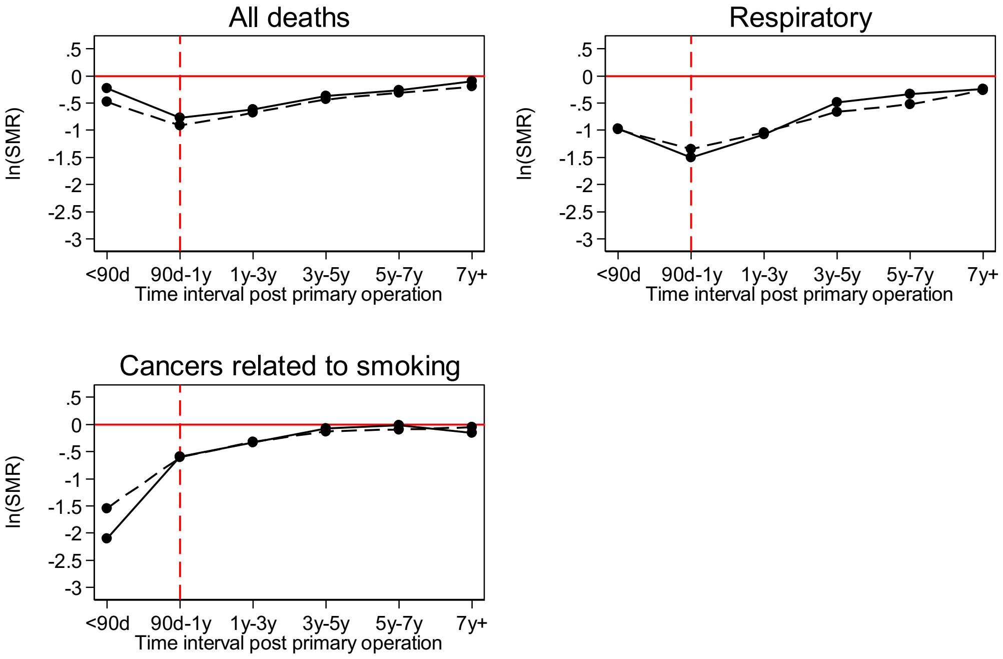

SMRS from Table 3: Hips Chemical thrombophylaxis: Aspirin (dashed line) vs Other/other combs of chemical prophylaxis (long dashed line) vs None (solid line)

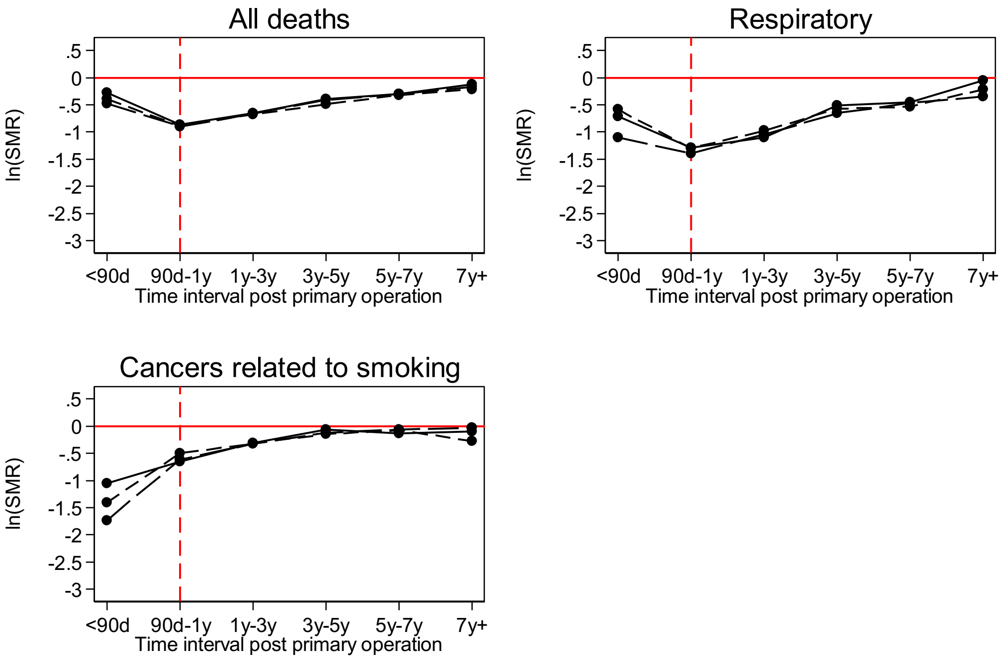

SMRs from Table 4: Hips Spinal anaesthetic (dashed line) vs GA (solid line) vs Spinal+GA (long dashed line) vs Other (dotted line)

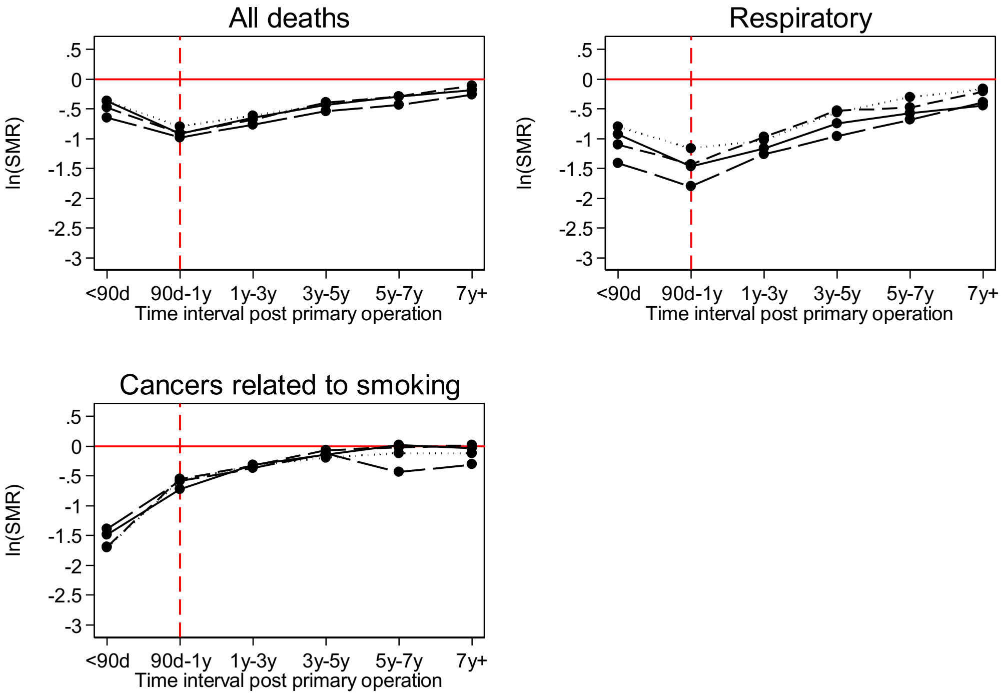

SMRs from Table 5: Hip Resurfacings (dashed line) vs. Uncemented hips (long dashed line) vs other hip types (solid line)

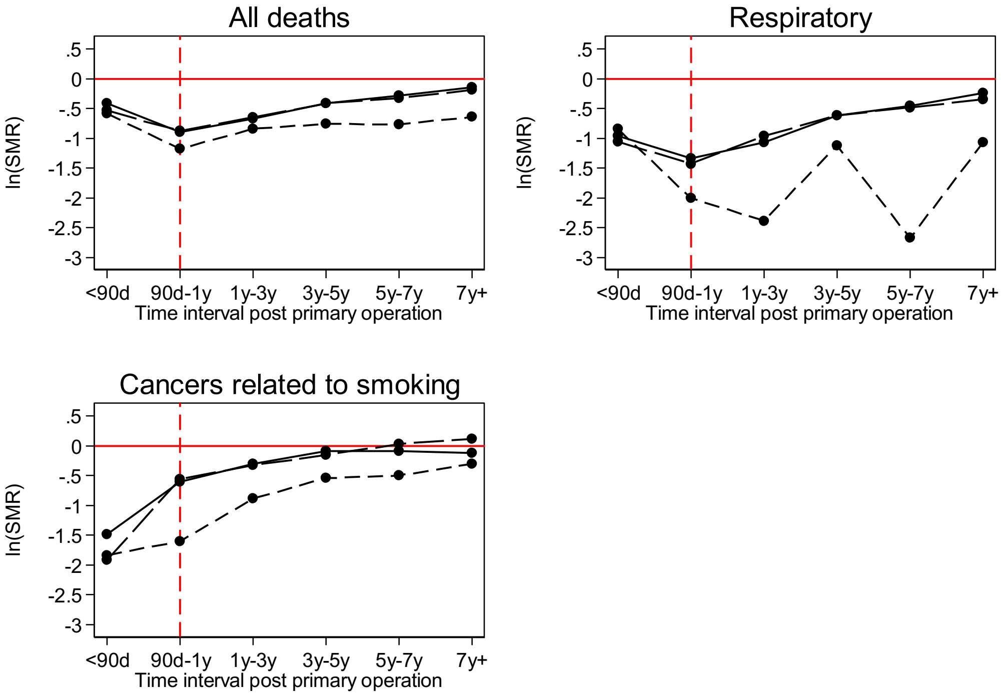

SMRs from Table 6: Unicondylar knees (dashed line) vs other knee types (solid line)

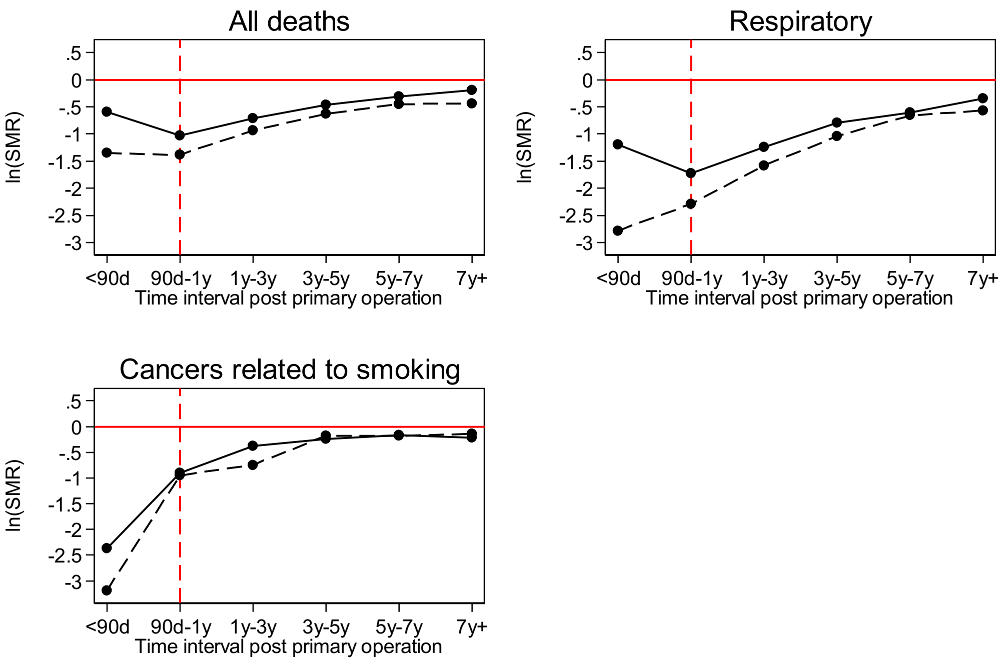

Supplementary Material Figures 2: Validation of the internal FPM models using just age and gender:

Initial exploratory work led us to use dfs of 5 and 4 (ie 4 and 3 'knots'), respectively, for the baseline hazards of hips and knees. These captured the patterns we had seen in earlier analyses, namely short term increases that had subsided by 90 and 45 days, subsequently increasing with time, reflective of normal mortality.

Mortality for men was higher than women and was increased with increased age at primary operation, but these effects of these factors changed significantly with time from the primary operation ($p=0{\cdot}006$, $p<0{\cdot}001$ respectively for gender and age for hips and $p<0{\cdot}001$, $p<0{\cdot}002$ for knees, likelihood-ratio tests). These time-varying effects were best captured using spline functions for gender and for age (as 4 restricted cubic splines) using dfs=1 and 4 for hips and df=2 and 2 for knees.

The figures below demonstrate our fitted models. The first show model-predicted hazard rates and cumulative mortality for men and women at 60, 70, 80 and 90 years at the time of their primary operation. The second compare the actual cumulative mortality (Kaplan-Meier curves) for men and women within 7 age-bands (shown with solid coloured lines) with the cumulative mortality as predicted by the model (black dotted lines). These illustrate that our models as described above for hips and knees fitted the data well.

Model estimated hazard rates (LHS) and cumulative probability of deaths (RHS) for males (M) and females (F) aged 60, 70, 80 and 90 years at operation.

(i) Hips:

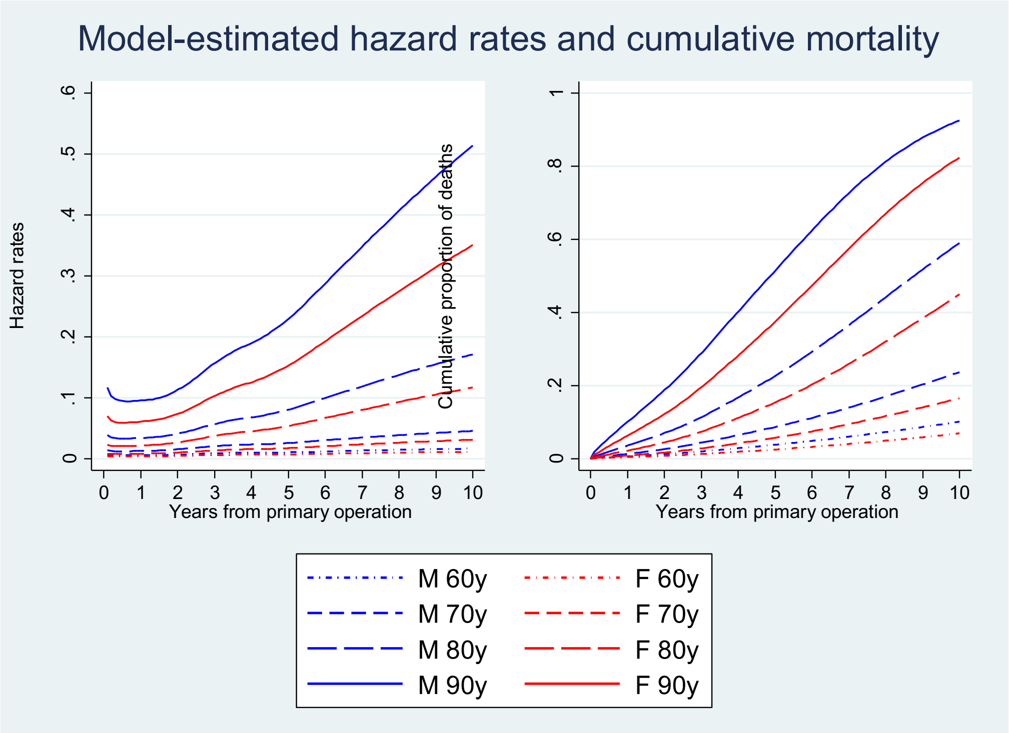

(ii) Knees:

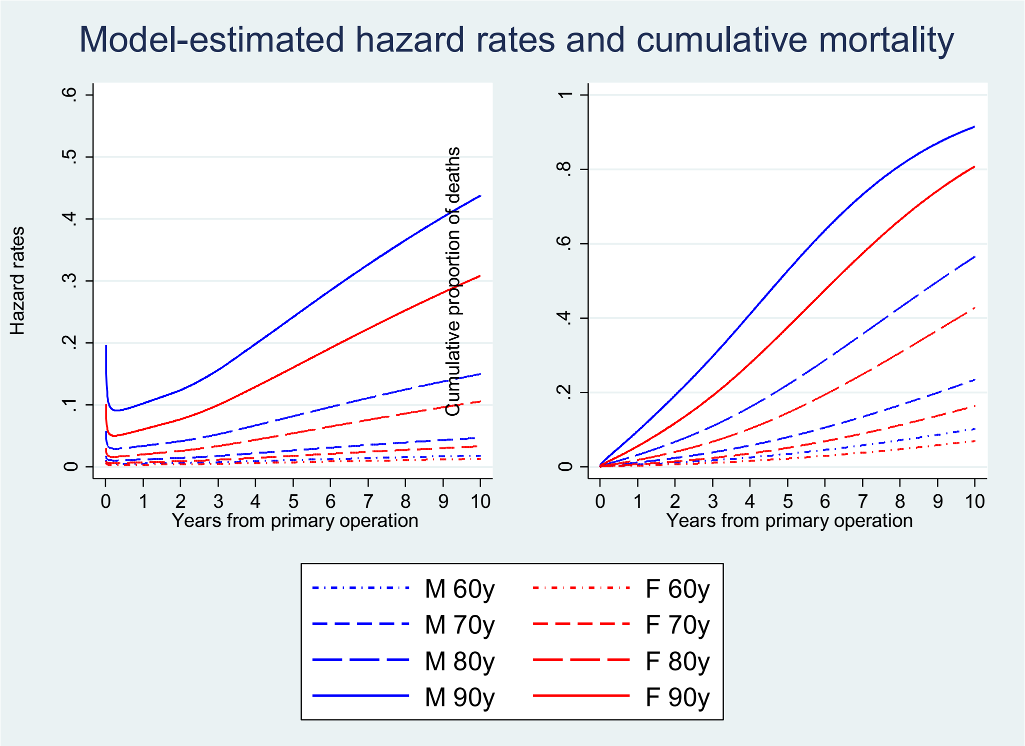

Kaplan-Meier estimates of cumulative mortality for 7 age groups (<55, 55-59, 60-64, 65-69, 70-74, 75-9 and 80+ years) for males (left side panel) and females (right side panel) separately.

(i) Hips

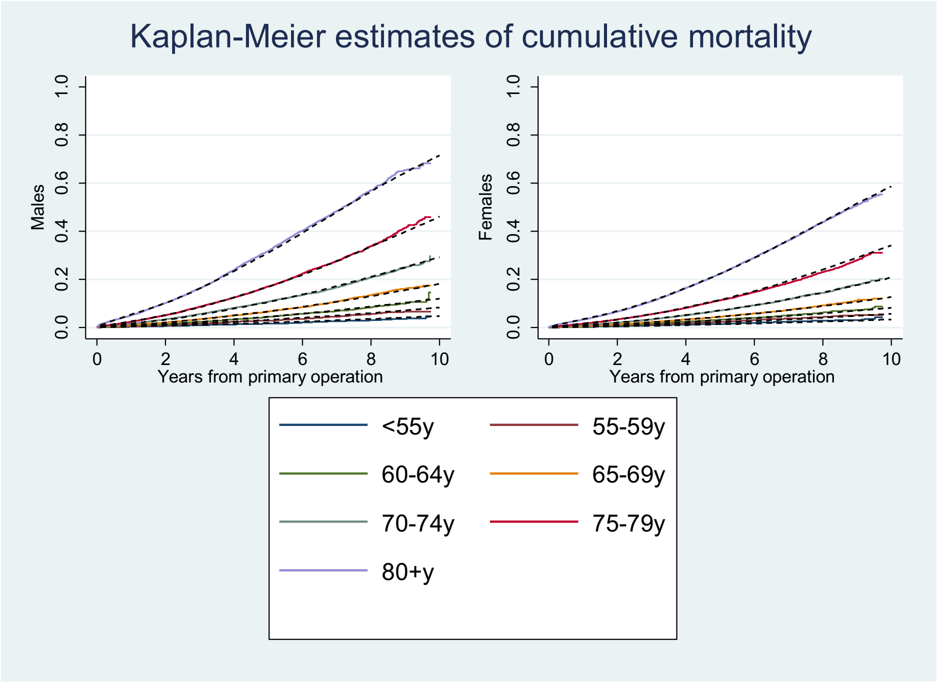

(ii) Knees

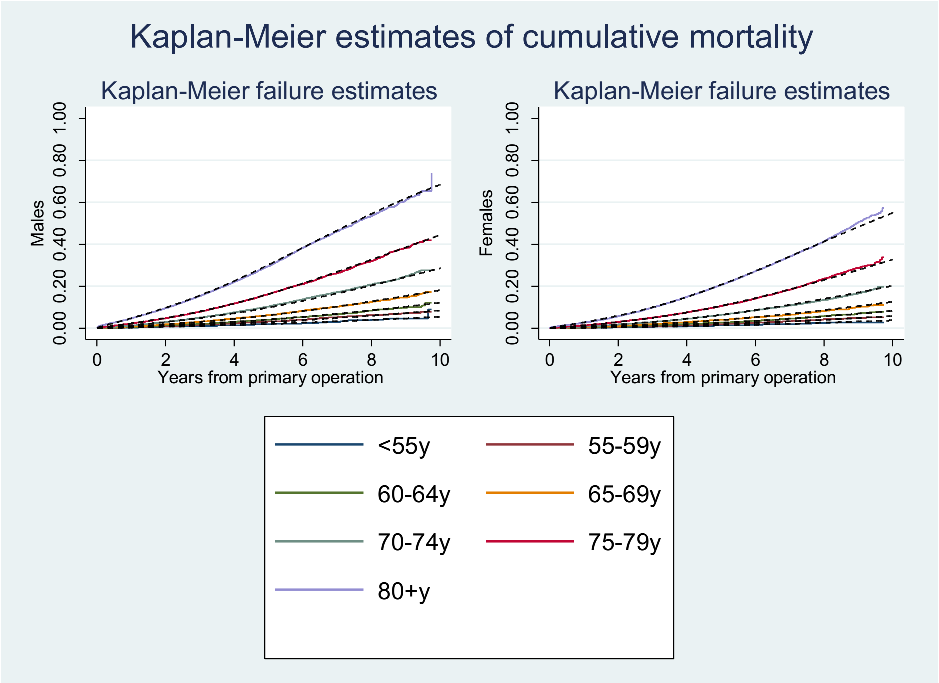

Supplementary Material Figures 3: Extending the internal (FPM) models to include other factors

Hips:

Grouped year of operation, ASA, surgical approach, anaesthetic used, mechanical prophylaxis, chemical prophylaxis and implant type (see S1(i) Table in the supplementary material) were all added to the model with gender and age (described fully in S6 above; n=411,481 hip primaries with complete information).

Year of operation, ASA and posterior approach had significant time-varying effects (p<0·001 for each, likelihood ratio tests) and modelled with df=1, 2 and 1 respectively (see S7 Fig (i) below and Fig 1 in the main text). Similarly, the effects of mechanical thromboprophylaxis and anaesthetic varied with time (p=0·011 and p=0·046) and each modelled with df=1 (Figs 2(a) and 3 in the main text). Initially chemothromboprophylaxis and hip type appeared not to vary with time overall (p=0·264 and p=0·412, each with df=1) but we found differences between the categories of each risk factor, and so allowed for the effects to vary (using df=1) to explore this (Fig 2(b) and Fig 4 in the main text).

The model-estimated HRs associated with year of operation and ASA are shown in Fig (i) below. For year of operation, the reference category (red line) is ‘2003-5’, i.e. the first three years of the registry data collection. The plot demonstrates that early mortality risk, i.e. mortality in the early phase after the operation, successively decreased for ‘2006-8’ (blue line) and ‘2009-12’ (black line). However this early advantage diminished with time from the primary operation. Likewise, compared with a reference category of ‘P1’ for ASA (red line), those with ASA scores ‘P2’ (green) and ‘P3’ (blue) and ‘P4/P5’ (black) at the time of the operation fared worse initially but the relative effects diminished with time from operation.

In Fig 1 of the main text, a posterior surgical approach (black line) was initially associated with a lower mortality than other approaches (red line). The effect was not lasting, and diminished with time from operation. The dotted lines indicate pointwise 95% confidence intervals (CI); they suggest no further advantage after 2-years, although it is not possible to precisely define the time when the effect becomes ‘non-significant’ as this is a fairly ‘blunt instrument’.

Fig 2 of the main text shows hazard rates ratios associated with mechanical and chemothromboprophylaxis. The left hand side of Fig 2(a) shows the HR associated with mechanical prophylaxis, compared with referent ‘no mechanical prophylaxis’ (red line). There was a relative decrease in mortality initially but this effect diminished with time. Results for the various chemical prophylaxis subgroups however differed (right hand side of Fig 2(b)). Compared with a referent group of ‘no chemoprophylaxis’ (red), ‘aspirin only’ (black) was associated with a reduction in mortality that persisted over the period observed. ‘Heparin + aspirin (brown dashed), was associated with an initial marked reduction that slowly receded; ‘Heparin only’ (blue), was associated with a less marked reduction that also slowly receded; a similar but again less marked effect was seem with ‘other chemoprophylaxis/other combinations’ (green). Pointwise 95% CIs, shown as dashed lines, are colour-coded by category.

Fig 3 in the main text shows hazard rate ratios associated with different forms of anaesthetic**.** Compared with general anaesthetic (GA), spinal anaesthetic was more advantageous early on but this effect diminished with time.

In Fig 4 in the main text**,** uncemented hips had lower mortality than cemented hips initially and this effect seemed to be time invariant, i.e. did not change with time. Hybrid and reverse hybrid fixation, however, were associated with higher mortality initially but these effects diminished with time. Resurfacings had markedly reduced mortality and the effect was time-invariant; this finding was still present with further adjustment for quintile of area deprivation and for comorbidity (figures not shown).

Knees:

Adding in year group, ASA, and knee type to the model with age and gender showed each of these factors to have time varying effects (p=0·004, p<0·001, and p<0·001 respectively).

Results for year group and ASA grade (using respective dfs=1 and 2) are shown below (S7 Figure (ii)). The early effects of year of surgery and ASA on early mortality diminished with time from the primary operation.

Main text Fig 5 shows the reduced mortality seen in UKR in the early period after surgery, this effect diminished with time; this initial advantage was lost more quickly with further adjustment for co-morbidity or social class (not shown).

Hazard rates ratios associated with year of operation and ASA grade for (i) hips (adjusting for time varying effects of age and gender, anaesthetic used, mechanical prophylaxis, chemical prophylaxis and implant type) and (ii) knees (adjusting for time varying effects of age, gender and implant type).

(i) Hips

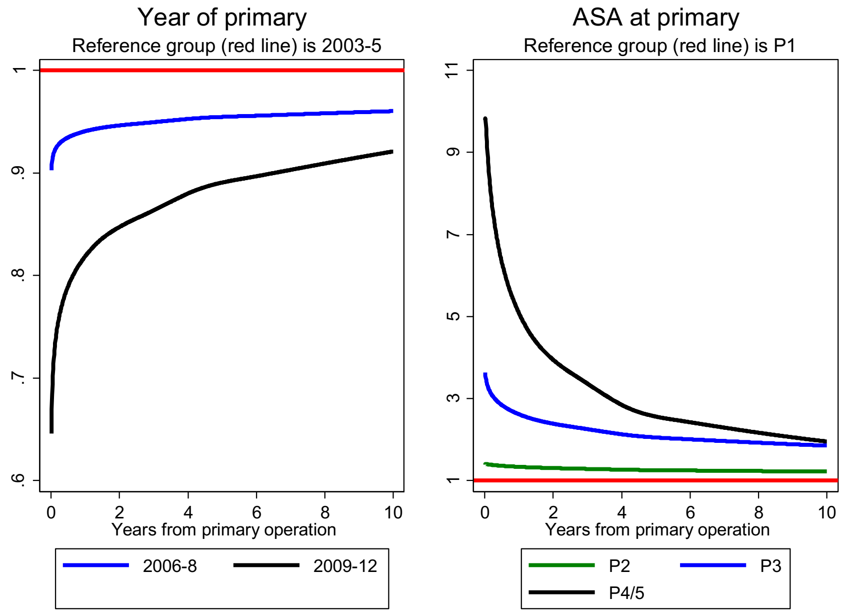

(ii) Knees

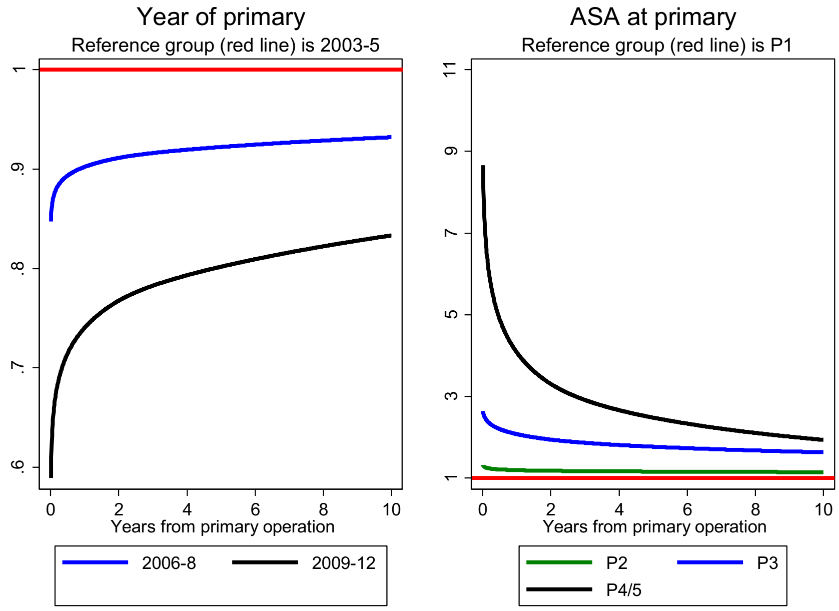

Supplementary Material Figures 4: Additional FPM analyses for hips – hip type/bearing

In a separate analysis, hip type was reclassified by subdividing each of the cemented, uncemented, and hybrid subgroups into metal-on-metal articulations vs. other bearing types (see S1 Table (ii) for the list of 'other' bearing subgroups: note 'other/unsure' subgroups were omitted from this analysis; n=403,573 with complete data). The reduced mortality shown in Figure 4 in the main text persisted in this analysis.

Hazard rates ratios associated with hip type/bearing (adjusting for time varying effects of age, gender, year of operation, ASA, surgical approach, anaesthetic used, mechanical prophylaxis and chemical prophylaxis)

'Cemented', 'uncemented', 'hybrid', 'reverse hybrid' and 'resurfacings' are shown respectively with red, black, brown, green and blue lines. Dashed lines indicate 'MoM', solid lines are 'other bearing surface's (see text). The reference group is 'Cemented non-MoM' and denoted by solid red line. CIs have been omitted for simplicity.

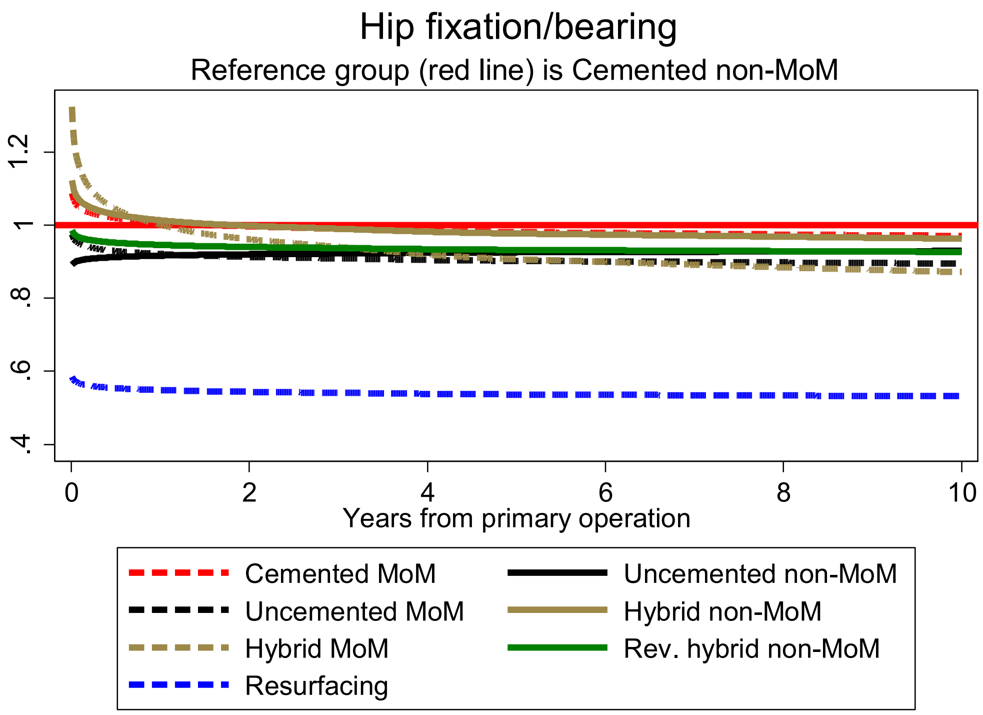

Supplementary Material Figures 5: Additional FPM analyses for hips – BMI

Finally, the BMI subgroup was added to the model with all variables (see S6**)**. The number of cases with full data available was much reduced (from 411,481 to 188,487). Whilst the effect of BMI did not change significantly with time (p=0.109), when modelled with df=1 (S8 Figure) there was an initial increased mortality in the very underweight (<19 kg/m^2) that diminished with time. An apparent reduced mortality in the overweight (26-30 kg/m^2) was only evident early on and its effect diminished with time.

Hazard rates ratios associated with BMI subgroups (adjusting for time varying effects of age, gender, year of operation, ASA, surgical approach, anaesthetic used, mechanical prophylaxis, chemical prophylaxis and implant type; n=188,487 with complete information)

The reference group is 'normal weight' (19-25 kg/m^2) and denoted by a red line. Underweight (<19 kg/m^2), overweight (26-30 kg/m^2) and obese (>30 kg/m^2) are shown respectively with solid blue, black and green lines, with corresponding 95% CIs shown as dashed lines

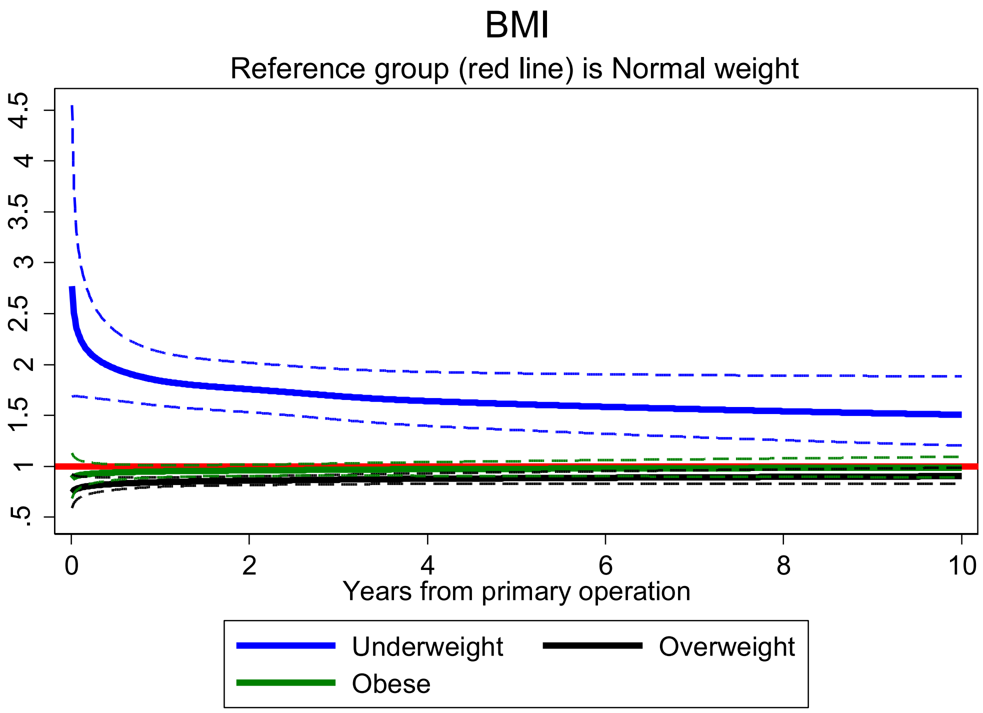

Supplement: Supplementary file 1 — Supplementary material [file 41598_2018_33314_MOESM1_ESM.pdf]
